# Observations on the Means of Reducing Strangulated Herniæ; in a Letter to Mr. R. W. Bampfield

**Published:** 1821-01

**Authors:** 


*Observations on the Means of Reducing Strangulated Herniæ; in a Letter to* Mr. R. W. Bampfield. By Dr. Sherwen.

I HAVE lately read, with much satisfaction, your paper in the Medical and Physical Journal of September last, on the advantage of long-continued horizontal position in an old and otherwise irreducible entero-epiplocele; which induces me to offer, through your hands, for the same publication, a short account of two cases of acute incarcerated herniæ, in which the successful treatment was perhaps not less owing to position than to the employment of other confessedly powerful means. But, before I enter upon the cases, permit me to remark, that, in my opinion, the great progress medical science, both general and particular, hath made of late years, may in a great measure be ascribed to the rapidity with which medical facts and information are now promulgated and propagated through the medium of periodical publications; and I know of none that has obtained a more extensive circulation than that in which your excellent paper appears. Heretofore, many a valuable fact was often confined to a particular medical man or his immediate medical friends; with one or more of whom such facts may have been buried and lost to the profession at large: whereas, now, no sooner does a valuable observation occur to any respectable member of the profession, or no sooner does such an one imagine he has made a valuable observation, than it finds its way into some periodical publication, where it either meets with approbation or is soon refuted by the keen eye of medical criticism. But to return.

I spent a considerable part of last summer at Enfield, where I was requested by Mr. Jessop, who had been more than twenty years the assistant of my successor Dr. Clarke, to visit a patient of his, then in the second day of a painful incarcerated inguinal hernia, which had resisted all his attempts at reduction. The patient, with an old and ill-adapted elastic truss, had been working hard in the hay-field, and probably drinking freely. His skin was hot, face flushed, and his pulse far from feeble; but, on account of his age, 75, he had objected to bleeding; which was now immediately performed, the tobacco-clyster, without loss of time, administered, and a large dose of calomel prescribed. The patient was now placed, with his head and shoulders resting on a mattress, upon the floor of the chamber, with his legs and thighs upon the bed, as high as the furniture would permit; and, in this position, napkins wetted with a refrigerating solution, which had been previously very properly applied, were ordered to be continued, and repeatedly changed during five or six hours. At the end of that time Mr. Jessop was directed to attempt reduction again; when he had the pleasure to find the contents of the tumor, about the size of a large clenched fist, slipped up at the first touch. The febrile symptoms, however, continued to run so high that he found it necessary to repeat the bleeding in the course of the night: and from that time the recovery was as rapid as could be expected.

Twenty-three years ago I found the same position produce the same good effect, without either bleeding or the tobacco-injection, but with the advantage of the cold application, in the treatment of a patient of the same age, in Tuckey-street; and I now take blame to myself for not having then communicated the result of the practice. The best apology I can offer is, that it was my intention to have done so through the medium of the Medical Spectator; the continuance of which work was impeded, first by a domestic affliction, and afterwards by the dreadful fire in Red Lion-passage, Fleet-street; where the greater part of the first edition of the three volumes was consumed.

In respect to this continued position, it must be evident that if the finger and thumb could be applied, within the abdomen, to that portion of intestine contiguous to the internal ring, the slightest effort would immediately retract a contiguous portion; which the rudest, the roughest, and, let me add, even the most judicious, pressure *ab extra*, with every advantage of scientific relaxation of muscles and ligaments, would in vain attempt. But, what is denied to the finger and thumb,* is perhaps afforded by due attention to this position.

Let one or more folded sheets be tied from one foot-post of the bedstead to the other, at proper heights. Cover these with blankets and pillows; and let the patient be so placed that his body shall rest partly on the neck and shoulders on other pillows in the bed, and partly by the legs hanging over the top of the highest folded sheet; or the legs may be placed over the arms of a sofa, with which most houses are provided.

In this position the whole contents of the abdomen gravitate towards the diaphragm; there is a constant, but gentle and almost imperceptible, tendency to retraction of the confined

---

* An intelligent medical friend remarks, "I should not be surprised if hereafter some enterprising surgeon of plain good sense (the best of all sense) should adopt your hint; and, by making an opening into the cavity of the abdomen, apply his finger and thumb in the manner you mention, and with facility effect the reduction." May it not be added, will the patient, by such an operation, undergo as much pain,—will he have as large a surface exposed,—or will the internal cavity of the abdomen be more subjected to the danger of inflammation from the admission of external air, than in the usual operation?—will there be as much danger of wounding either the epigastric artery or the spermatic vessels? If none of these dangers will be augmented, but all of them lessened, why should he hesitate to adopt the hint? The practical operating surgeon may probably advance unanswerable objections: none occur at present to the writer,—old adhesions excepted.

portion of intestine from the herniary tumor: and even the efforts to vomit, (which in the common position are injurious,) and the inverted peristaltic motion, become auxiliaries. A few hours of this position, with an assiduous application of the cold napkins, will retard the progress of inflammation, and, with the other well-known means, give nature fair play.

I cannot help taking this opportunity of remarking, that I think our anatomical and surgical teachers are inclined rather too much to urge the necessity of early operation in these cases. It may be difficult to point out the precise and most proper time for decision, as that must always be left to the judgment and experience of the surgeon: every prudent one will, if possible, obtain the sanction and concurrence either of a professional friend or senior practitioner. A bold and dextrous operator, fond of the knife, may be apt to operate too soon: a timid or an unskilful one, too late. Both may, I believe, be often relieved from embarrassment, by attention to the position here recommended; which another case in point may further explain.

A gentleman, labouring under painful, acute, incarcerated hernia, called in the assistance of one whom I know to be a very skilful and experienced surgeon; who, after every proper attempt at reduction, urged the necessity of an operation, which the patient resisted on this ground, that his family had been successfully attended by the junior partner, then absent, for whom he would wait. The junior partner arrived in the evening, when, on failure of reduction, the operation was again mutually urged. But it was now found that the previous deferring of compliance on account of the absence of the junior partner, had been merely pretence; the patient having determined, in his own mind, not to submit to any operation. On repeating the visit next morning, the two surgeons had the pleasure to find their patient in a state of perfect security,—spontaneous reduction having taken place during the night.

I could here add other cases,

> Quæque ipse miserrima vidi
> Et quorum pars magna fui,—

in which some patients have been lost, who, I am now much inclined to believe, might have been preserved, had I more early had resource to the method I am here recommending.

It may, perhaps, be said there is nothing new in the proposal, —other surgeons having been, from time immemorial, occasionally in the habit of placing the patient's legs over the shoulders of an assistant, with his head hanging down. I know that this has often been done; and by so doing, and sprinkling the tumor with cold water, I have myself succeeded in dangerous cases: but the difference is great between placing a patient in this position during a few minutes, while reduction by the hands is attempted, with all the abdominal muscles in a state of tension; and the gradual, slow, and easy effect of quietude, and the uninterrupted efforts of nature, whilst the patient is in a state of relaxation and comparative repose.

I have not the honour, Sir, of your acquaintance; but your valuable paper is the parent of mine, which, but for the stimulus of yours, might have continued, *de die in diem*, in a state of intended preparation and publication.

*Bath; Oct. 30th,* 1820.

P. S.—On this occasion it may be right to republish what I apprehend may have already appeared in the Medical and Physical Journal, —viz. the popular practice in Russia in cases of incarcerated hernia, as mentioned by John Conrad Hiltebrand. "A pot, capable of holding several pints, is applied in the manner of a cupping-glass to the abdomen, previously rubbed with oil or soap. The parietes and bowels are thus drawn (with pain) into the pot, and the parts contained in the rupture into the abdomen." He asserts that many, under very desperate appearances have been thus cured. He might have added, that it was the dictate of plain good sense. The cupping may, with the least inconvenience, be performed by dropping a piece of silver paper wetted with rectified spirit, and lighted, into a large bell-tumbler, or any other vessel properly capacious.

